# Football-related injuries are the major reason for the career end of professional male football players

**DOI:** 10.1007/s00167-021-06684-8

**Published:** 2021-08-09

**Authors:** Matthias Koch, Martin Klügl, Borys Frankewycz, Siegmund Lang, Michael Worlicek, Daniel Popp, Volker Alt, Werner Krutsch

**Affiliations:** 1 Department of Trauma Surgery, University Medical Centre Regensburg, Regensburg, Germany; 2 herz:bewegt - Praxis für Kardiologie und Sportmedizin, Bahnhofstraße 19, 94315 Straubing, Germany; 3 SportDocsFranken, Nuernberg, Germany

**Keywords:** Retirement in football, Professional football player, Injury, Osteoarthritis, Knee injury, Football, Soccer

## Abstract

**Purpose:**

Little is known about the consequences of injuries on professional male football players’ career and retirement period. The aim of this study is to investigate the impact of injuries that male professional football players endure during their career, reasons for the end of their career and the post-career phase of retirement.

**Methods:**

In a retrospective cross-sectional cohort study, retired male professional football players of the German Bundesliga were investigated by a standardised questionnaire to analyse the history of injuries sustained during their professional football career, the reasons for ending their career, their current health status and their suggestions for future prevention strategies.

**Results:**

Most of the 116 analysed players (*n* = 73 (62.9%)) stated an injury as the reason for ending their professional career. Relevant injuries were mainly located in the lower extremities (*n* = 587 (61.3%)) with a focus on the knee (*p* < 0.001) and ankle (*p* < 0.001). A significant majority of the participants who had retired due to injury described degenerative symptoms, such as pain or instability, and were diagnosed with osteoarthritis after retirement (*p* < 0.001). These players had also often been affected by symptoms of depression during their career, which had decreased significantly after retirement. Moreover, players who had not retired due to injury had significantly better overall health status and quality of life after retirement.

**Conclusion:**

Football-related injuries have a significant impact on the career end of professional male football players and their health status after retirement. Future prevention strategies need to particularly address injuries to the knees and ankles and to implement measures for preventing osteoarthritis after retirement.

***Level of evidence*:**

Level III

## Introduction

Football is the most common type of sports worldwide and is associated with a high incidence of injuries [1, 2]. Many studies have analysed injury patterns with regard to the different activity levels or training vs. match [1–3]. Overall, professional football players have a high risk of sustaining injuries [1, 4]. Most injuries occur during matches and affect the lower extremities [1, 3, 5, 6]. Besides the consecutive time loss [7], the negative effect on team performance [5], and the possible long-term consequences, such as an increased risk of osteoarthritis [8], football-related injuries may influence a player’s career and quality of life [5]. Thus, many studies focus on specific treatment and rehabilitation strategies to restore the sports abilities of athletes as soon as possible to reduce the risk of long-term impairment.

Yet, little is known about the real impact of football injuries on the career end and the retirement period of professional football players.

Thus, the aim of this study is to investigate the impact of injuries of professional football players during their career, related reasons for the end of their career and their retirement with a special focus on the health status after the end of the career. The knowledge about mid-term and long-term consequences of football-related injuries obtained in this study will help to improve the risk assessment for teams and players and enable the adequate development of prevention strategies to be already implemented during the active career of professional football players.

The hypothesis of this study is that not only the career course of professional football players in terms of timing the end of their career but also sports activities and the health status after their career is dramatically influenced by football-related injuries.

## Materials and methods

The design and methods of this study were approved by the local Ethical Committee (University of Regensburg; Ref. 10-101-0075). A cross-sectional cohort study design was chosen for analysing injuries that caused the end of professional football careers and the consecutive health status of retired professional male football players. The study was conducted in cooperation with the German labour union of professional football players (Vereinigung der Vertragsfußballspieler, VDV), which takes care of active and retired professional football players in Germany and acts as a counsellor for players with legal, health and economic issues. The VDV distributed the study guidelines and a questionnaire to a series of 200 former elite male football players and collected written consent forms for participation in the study. The players reported injury diagnoses and additional medical data based on the information given by their physicians.

Professional football players were defined as players who had an elite contract with a team of the 1st or 2nd Bundesliga, which represent the two highest playing levels in Germany. Further inclusion criteria were a minimum of one official match in a team of these two professional football leagues. Exclusion criterion was an incomplete questionnaire.

The questionnaire included information about the participant’s characteristics, career details, history of football-related injuries, reasons for ending the career, the current health and activity status as well as a retrospective assessment of the prevention strategies available during the player’s professional football career. The questions are shown in the corresponding tables. Overall, the questionnaire was based on standardised injury surveys previously implemented by Fuller et al. [9] and Hägglund et al. [10].

The participants were divided into two groups, depending on the reason for ending their professional career. The first group (group 1) consisted of players who had retired due to one or more injuries, and the second group (group 2) included all players stating that they had no medical reason for ending their professional football career.

## Statistical analysis

Statistical analysis was performed using SPSS^®^ (Version 25, IBM, Armonk, NY, USA). Because of the absence of a specific primary endpoint, no a priori sample size calculation was carried out. The number of included patients was based on the data available from the VDV. Data are presented as mean ± standard deviation (SD) and 95% confidence interval (CI) (continuous data) or absolute and relative frequencies (categorical data). Continuous data were compared between the two groups using the Mann–Whitney *U* test. Categorical data were assessed using cross tabulation methods with the *χ*^2^ test. A probability (*p*) value of ≤ 0.05 was considered to be significant.

## Results

*N* = 126 (63.0%) retired players returned the questionnaire, of whom *n* = 10 had to be excluded from the study because of an incomplete questionnaire. In general, the end of the professional football career was followed by a significant increase in mean BMI (*p* ≤ 0.001, Table 1). The epidemiological data did not significantly differ between the two groups (Table 1).

**Table 1:** Anthropometric and football-specific data of former professional football players

		Total *n* = 116	Group 1 End of career due to injury *n* = 73	Group 2 End of career due to non-medical reasons *n* = 43
Age at end of professional career	Mean ± SD (95% CI) [y]	32.2 ± 4.2 (31.5–33.0)	31.8 ± 4.1 (30.8–32.7)	33.1 ± 4.2 (31.8–34.4)
Duration of complete football career	Mean ± SD (95% CI) [y]	25.6 ± 4.5 (24.8–26.4)	25.0 ± 4.7 (23.8–26.1)	26.6 ± 4.0 (25.4–27.8)
Official matches				
1–50	*n* (%)	16 (13.8)	4 (5.5)	12 (27.9)
51–100	*n* (%)	4 (3.4)	3 (4.1)	1 (2.3)
101–200	*n* (%)	21 (18.1)	19 (26.0)	2 (4.7)
201–300	*n* (%)	27 (23.3)	23 (31.5)	4 (9.3)
> 300	*n* (%)	48 (41.4)	24 (32.9)	24 (55.8)
Favourite striking leg				
Right	*n* (%)	92 (79.3)	55 (75.3)	37 (86.0)
Left	*n* (%)	24 (20.7)	18 (24.7)	6 (14.0)
Position on field				
Goalkeeper	*n* (%)	12 (10.3)	5 (6.8)	7 (16.3)
Defender	*n* (%)	34 (29.3)	22 (30.1)	12 (27.9)
Midfielder	*n* (%)	47 (40.5)	32 (43.8)	15 (34.9)
Striker	*n* (%)	23 (19.8)	14 (19.2)	9 (20.9)
Body-mass-index				
During the career	Mean ± SD (95% CI)	22.7 ± 4.5 (21.9–23.5)	23.2 ± 3.0 (22.5–23.9)	22.0 ± 6.2 (20.1–23.9)
After the career	Mean ± SD (95% CI)	25.5 ± 1.8 (25.2–25.9)*	25.6 ± 1.7 (25.1–26.0)	25.5 ± 2.0 (24.8–26.1)*
Educational degree				
Main school	*n* (%)	7 (6.0)	3 (4.1)	4 (9.3)
Middle school	*n* (%)	20 (17.2)	14 (19.2)	6 (14.0)
Vocational school	*n* (%)	51 (44.0)	32 (43.8)	19 (44.2)
Academic high school	*n* (%)	20 (17.2)	13 (17.8)	7 (16.3)
College for further education	*n* (%)	12 (10.3)	9 (12.3)	3 (7.0)
University degree	*n* (%)	6 (5.2)	2 (2.7)	4 (9.3)

^*^Significantly increased compared to duration of professional career (*p* ≤ 0.05)

The most significant reason for football players to end their professional career were medical issues (medical vs. non-medical reasons for ending the career: *p* ≤ 0.001) followed by age-related issues as the main non-medical reason (Table 2).

**Table 2:** Reasons for the end of the professional football career

Reasons for the end of the career	Total *n* = 116 *n* (%)	Group 1 End of career due to injury *n* = 73 *n* (%)	Group 2 End of career due to non-medical reasons *n* = 43 *n* (%)
Medical	73 (62.9)	73 (100)	–
Acute injury	45 (38.8)	45 (61.6)	–
Chronic injury/series of injuries	28 (24.1)	28 (38.4)	–
Non-medical	43 (37.1)		
Age	31 (26.7)		31 (72.1)
Alternative job	8 (6.9)		8 (18.6)
Personal reasons	4 (3.4)		4 (9.3)

Players in group 1 had a significantly higher number of injuries per player during their professional football career than players in group 2 (*p* < 0.001). In group 1, knee injuries (*n* = 155 (23.8%) vs. *n* = 41 (13.3%); *p* < 0.001) and ankle injuries (*n* = 67 (10.3%) vs. *n* = 18 (5.8%); *p* < 0.001) were significantly associated with retirement due to injury (Table 3).

**Table 3:** Injury occurrence and injured body regions of former professional football players

		Total *n* = 116	Group 1 End of career due to injury *n* = 73	Group 2 End of career due to non-medical reasons *n* = 43
Number of injuries	*n*	958	650	308
Number of injuries per player	mean ± SD	8.3 ± 3.6	8.9 ± 3.7 *	7.2 ± 3.2
Head	*n* (%)	190 (19.8)	124 (19.1)	66 (21.4)
Upper Limb	*n* (%)	141 (14.7)	92 (14.2)	49 (15.9)
Shoulder	*n* (%)	43 (4.5)	31 (4.8)	12 (3.9)
Elbow	*n* (%)	10 (1.0)	9 (1.4)	1 (0.3)
Finger	*n* (%)	88 (9.2)	52 (8.0)	36 (11.7)
Trunk	*n* (%)	15 (1.6)	9 (1.4)	6 (1.9)
Spine	*n* (%)	25 (2.6)	16 (2.5)	9 (2.9)
Lower Limb	*n* (%)	587 (61.3)	409 (62.9)	178 (57.8)
Thigh	*n* (%)	232 (24.2)	145 (22.3)	87 (28.2)
Knee	*n* (%)	196 (20.5)	155 (23.8)*	41 (13.3)
Lower leg	*n* (%)	21 (2.2)	14 (2.2)	7 (2.3)
Ankle	*n* (%)	85 (8.9)	67 (10.3)*	18 (5.8)
Foot	*n* (%)	53 (5.5)	28 (4.3)	25 (8.1)

^*^Significantly increased compared to the other group (*p* ≤ 0.05)

After retirement, the level of overall sports activities differed significantly between the two groups (*p* = 0.03). With regard to football activities, however, significantly fewer players continued playing football after retirement in group 1 than in group 2 (*p* = 0.003). In addition, players in group 1 more frequently required assistive equipment, such as taping, bandages or orthosis (n.s.), and analgesics (*p* = 0.01), in accordance to the description of symptoms, such as pain (*p* < 0.001) or instability (*p* < 0.001). The group of retirement due to injury was characterised with an almost twice as high prevalence of osteoarthritis (*n* = 58 (79.5%) vs. *n* = 18 (41.9%); *p* < 0.001), especially regarding knee osteoarthritis (*p* = 0.001) (Table 4). Overall, the diagnosis of osteoarthritis significantly correlated with the typical degenerative symptoms as described above (pain (*p* < 0.001), instability (*p* < 0.001) and effusion (*p* = 0.005) (Table 4).

**Table 4:** Activity and health status after the end of the professional football career

	Total *n* = 116 *n* (%)	Group 1 End of career due to injury *n* = 73 *n* (%)	Group 2 End of career due to non-medical reasons *n* = 43 *n* (%)
Sports activities	101 (87.1)	59 (80.8)*	42 (97.7)
Playing football			
No	29 (25.0)	25 (34.2)*	4 (9.3)
Yes	87 (75.0)	48 (65.8)*	39 (90.7)
Assistive equipment	16 (13.8)	13 (17.8)	3 (7.0)
Symptoms			
Pain	81 (69.8)	60 (82.2)*	21 (48.8)
Instability	49 (42.2)	42 (57.5)*	7 (16.3)
Effusion	43 (37.1)	30 (41.1)	13 (30.2)
Diagnosed osteoarthritis	76 (65.5)	58 (79.5)*	18 (41.9)
Hip	13 (11.2)	11 (15.1)	2 (4.7)
Knee	51 (44.0)	41 (56.2)*	10 (23.3)
Ankle	40 (34.5)	27 (37.0)	13 (30.2)
Shoulder	5 (4.3)	3 (4.1)	2 (4.7)
Using analgesics	26 (22.4)	21 (28.8)*	4 (9.3)

^*^Significant differences between group 1 and group 2 (*p* ≤ 0.05)

Players in group 1 were also significantly more often affected by symptoms of depression than players in group 2 (*p* = 0.002). After retirement, the overall prevalence of depression had significantly decreased (*p* = 0.04), but the rate remained higher in group 1 (n.s.). Players in group 1 had also significantly more often been afraid of an injury that could cause the end of their professional career (*p* < 0.001) and were significantly more negative and emotionally overwhelmed when they had to give up playing football professionally (*p* < 0.001). In addition, players in group 2 had significantly more often planned their future profession after retiring from professional football (*p* = 0.050) and had slightly fewer problems in the transition from their football career to their current employment (n.s.). Accordingly, players who did not have to end their career due to injury were significantly more satisfied with their health (*p* < 0.001) and quality of life (*p* < 0.008) after retirement (Table 5).

**Table 5:** Psychological aspects of football-related injuries and retirement

		Total *N* = 116	Group 1 End of career due to injury *N* = 73	Group 2 End of career due to non-medical reasons *N* = 43
Depression				
During career	*n* (%)	56 (48.3)^§^	46 (63.0)*	10 (23.3)
After retirement	*n* (%)	5 (4.3)	4 (5.5)	1 (2.3)
Fear of a career-ending injury during career	*n* (%)	45 (38.8)	39 (53.4)*	6 (14.0)
Negative emotions at the end of the career	*n* (%)	40 (34.5)	34 (46.6)*	6 (14.0)
Prepared for future professional career at the end of career	*n* (%)	59 (50.8)	32 (43.8)*	27 (62.8)
Problems in the transition from professional career to current employment	*n* (%)	28 (24.1)	21 (28.8)	7 (16.3)
Football-related employment after professional career	*n* (%)	62 (53.4)	45 (61.6)	17 (39.5)
Subjective evaluation of the current health condition	Mean ± SD (95% CI)	2.9 ± 0.7 (2.8–3.1)	3.2 ± 0.7 (3.0–3.3)*	2.6 ± 0.7 (2.4–2.8)
Subjective evaluation of the current quality of life	Mean ± SD (95% CI)	2.6 ± 0.8 (2.5–2.8)	2.8 ± 0.8 (2.6–3.0)*	2.4 ± 0.7 (2.2–2.6)

Subjective evaluation of the current status was graded on a scale from 1 (excellent) to 5 (weak) (2–very good; 3–good; 4–reduced)

*Significant differences between group 1 and group 2 (*p* ≤ 0.05)

^§^Significant differences between career and retirement status (*p* ≤ 0.05)

Retrospective evaluation of the professional career showed that players in group 2 had been significantly more satisfied with the medical care (*p* = 0.001) and subjective health conditions (*p* < 0.001) during their professional career.

Adapted training concepts and improved medical education to optimize injury prevention was highly desired by both groups (Table 6).

**Table 6:** Subjective career analysis and suggestions for injury prevention

		Total *N* = 116	**Group 1** End of career due to injury *N* = 73	**Group 2** End of career due to non-medical reasons *N* = 43
Retrospective evaluation of aspects during the professional football career				
Medical care	Mean ± SD (95% CI)	2.5 ± 1.2 (2.3–2.8)	2.8 ± 1.2 (2.5–3.1)*	2.1 ± 1.0 (1.8–2.4)
Subjective health condition	Mean ± SD (95% CI)	2.4 ± 1.0 (2.2–2.6)	2.8 ± 0.9 (2.5–3.0)*	1.9 ± 1.0 (1.6–2.2)
Success in sports	Mean ± SD (95% CI)	2.4 ± 1.0 (2.2–2.6)	2.8 ± 0.9 (2.2–2.7)	1.9 ± 1.0 (1.9–2.5)
Relationship with the team	Mean ± SD (95% CI)	2.3 ± 1.0 (2.1–2.5)	2.3 ± 1.1 (2.1–2.6)	2.2 ± 0.9 (1.9–2.4)
Active media work	Mean ± SD (95% CI)	2.7 ± 1.0 (2.5–2.9)	2.8 ± 1.1 (2.6–3.1)	2.5 ± 0.8 (2.3–2.8)
Emotional distress	Mean ± SD (95% CI)	2.4 ± 1.1 (2.2 –2.6)	2.4 ± 1.1 (2.1 –2.6)	2.4 ± 1.1 (2.0 –2.7)
Most recommendable strategy for injury prevention				
Training concept	*n* (%)	80 (69.0)	52 (71.2)	28 (65.1)
Equipment	*n* (%)	41 (35.3)	25 (34.2)	16 (37.2)
Policy	*n* (%)	15 (12.9)	10 (13.7)	5 (11.6)
Medical education	*n* (%)	68 (58.6)	45 (61.6)	23 (53.5)

Retrospective evaluation of different aspects during professional football career was graded on a scale from 1 (excellent) to 5 (weak) (2–very good; 3–good; 4–reduced)

*Significantly higher compared to other group (*p* ≤ 0.05)

## Discussion

The most important finding of the present study was the high number of former football players who had to end their professional career because of a football-related injury. The literature describes high incidences of football-related injuries, especially in professional football, ranging between 6.6 and 9.1 injuries per 1000 h football-related activity [1, 2, 11]. The current research is mainly focused on aspects, such as return-to-play [12–14] or the risk of developing osteoarthritis [8, 15, 16]. However, the literature lacks data on football-related injuries causing the end of professional careers and on the consecutive health status of former professional football players.

The present study shows for the first time that injuries are the most frequent reason for the end of professional football careers (*p* < 0.001). Analysis of the injury history showed a significantly higher number of injuries per player (*p* = 0.001) was detected for players who had to retire due to injury in comparison to players who retired for non-medical reasons. Even if injury location was distributed almost in accordance with the literature with focus on the lower extremities, as well as head injuries [4, 5, 7, 17], there were significant differences between the two study groups. Especially knee and ankle injuries were significantly more common sites of injury associated with a medically caused retirement. The degenerative character and the occurrence of such injuries in professional football are well known but frequently trivialised [8, 15, 16, 18]. The occurrence of symptoms, such as pain or joint instability, also significantly correlates with the development of osteoarthritis, especially in the knee joints. The increased injury incidence per joint as well as the risk of osteoarthritis were shown by Drawer and Fuller almost two decades ago in 2001 [19], when they investigated former professional football players registered with the English Professional Footballers' Association. The most common injuries leading to early retirement were also located in the knee joints concerning both, acute as well as chronic injuries. In addition, osteoarthritis in context of football-related injuries also predominantly affected the knee joints, followed by the ankle and hip joints. The findings of the present study show that the joints in the lower extremities are still the body sites most affected by injuries, just as 20 years ago. Although Ekstrand et al. described a decreasing injury rate in men’s professional football over the past 18 years, the data suggest that there is a persisting limitation of current prevention strategies [11]. This finding highlights the continuous medical necessity of more focused prevention strategies and rehabilitation programmes after injuries. Despite the implementation of many preventive measures in recent years and the ongoing focus on this topic in current research [20, 21], from a medical point of view, these measures have to be critically re-evaluated and eventually improved.

The prevalence of osteoarthritis in the group of former professional football players who did not end their career due to injury was clearly increased (*n* = 18 (41.9%)) in comparison to a non-professional sport population with a prevalence ranging between 19.0% and 28.0% [22, 23]. This may be explained by a higher frequency of injuries to the lower extremities in combination with an increased load on the lower extremities during professional football career [22, 24, 25]. Ultimately, professional football seems to be a considerable risk factor for the development of osteoarthritis [15, 26].

Another important focus of this study was the evaluation of the overall health status of former professional football players who had retired due to injury. Besides the physical health status of the former players, the present analysis included their psychological condition after retirement as well as their current health status. Although just a few former players indicated to be affected by depression, almost half of the football players reported to have felt depressive during their professional football career. Here, significant differences were found between the two study groups. Because episodes of depression were more prevalent in players who had ended their career due to injury, a correlation of psychological health issues with the incidence of injuries can be assumed. Therefore, addressing psychological health issues and raising adequate psychological health awareness among active players may be one pillar of injury prevention. In contrast to these findings, earlier studies evaluating former international professional football players with regard to symptoms of depression reported a rate of 35–39% of affected players and a lower rate during their active career [27, 28]. However, a decrease of depressive symptoms was also described by Prinz et al. who compared the active career with the retirement phase of elite female football players [29]. The differences found may be explained by different “affection types”. A significant number of players who retired due to injury reasons, were already afraid of sustaining an injury that might cause the end of their career while still playing football professionally. Such an “anxious type” of player may consequently be overwhelmed by ultimately having to retire due to injury, whereas the “non-anxious type” of player may be able to cope better with the consequences of an injury. This supposition can be supported by the fact that the players in group 2 were better prepared for the end of their career and had fewer problems in the transition period between their retirement from football and their current employment. In addition, players in group 2 rated their current health status and quality of life as significantly better than players in group 1. These results are also in accordance with findings in the current literature. When investigating the context of retirement and the health status after retirement, both Filbay et al. and Moreira et al. found in their review a correlation between inferior quality of life or life satisfaction and involuntary retirement from sports [30, 31]. Because the prevalence of symptoms of depression was already significantly higher during the active career of players in group 1, it can be hypothesised that these players had a depressive disposition that is correlated to distinctive stress management and trauma processing [32, 33]. Such depressive disposition may contribute to low physical health and proneness to injury. Recent research has shown a correlation between psychological health disorders (anxiety/depression) and severe injuries as well as between distress and severe injuries in football players [34–37]. After retirement due to injury, the pressure to perform may decrease together with the symptoms of depression. Such health aspects and postulations, however, need to be clarified in more specific surveys.

Finally, the overall pattern of the health status of former elite football players emphasises the special need for appropriate prevention strategies to optimise primary injury prevention and to reduce the number of career-threatening injuries. In this context, affected players particularly highlighted the importance of adapting training concepts and medical education for more effective injury prevention. Both aspects are already known as relevant factors for successful prevention programmes [11, 38–40]. Even structured generalised warm-up programmes may reduce common injuries by about one third. In addition, specific injuries, such as anterior cruciate ligament injuries or ankle injuries, may be prevented by additional proprioceptive training concepts [38, 40–42]. However, prevention programmes may only significantly reduce injury rates when they are consequently implemented in the training routine as shown by Krutsch et al. [43]. Yet, such implementation requires a high degree of willingness of both football players and coaches [44]. McKay et al. found not only limited injury awareness among coaches and players but also a significant gap in understanding injury prevention [40]. Therefore, medical education is highly relevant for effective prevention strategies. In addition, focused psychological health research and awareness strategies should be facilitated.

Overall, the results of the present study provide relevant knowledge about the most important injuries during professional male football careers, which appear to have an impact on their termination and associated long-term consequences. The information gained in this study serves as a basis for the development of specific prevention strategies at this playing level.

Despite the strength of the present study and its unique data collection, the study has some limitations that should be considered in the interpretation of its results. Despite the collaboration with the German labour union of professional football players, the overall sample size of this study is small. In addition, the retrospective study design has to be considered limitative regarding the completeness of injuries sustained by professional football players. Retired players are probably not able to report the exact occurrence of each of their injuries sustained during their football career, especially of minor injuries without any relevant time-loss. However, the objective of this study was the analysis of injuries leading to retirement of the player. Such injuries are typically associated with relevant periods of absence from sports and require surgical treatment; therefore, these types of injury are usually well remembered by the players and sufficiently documented by the responsible physicians as well as by the VDV. Despite all these limitations, the data obtained by this study provide a basis for future prospective studies with larger study populations.

## Conclusion

Football-related injuries are not only the most significant reason for the end of a professional football career but also lead to a significantly higher prevalence of osteoarthritis and associated symptoms. Psychological aspects, such as depression, seem to have a significant impact on players during their football career, and the prevalence of depression significantly decreases after retirement. Football players who end their professional career for non-medical reasons rate their health status and quality of life after retirement significantly better than players who had to retire due to injury. The overall health status of former elite football players emphasises the requirement of appropriate injury prevention strategies.
